# Influence of GLP1 receptor *rs6923761* and *rs761387* genetic variants on oral semaglutide response in patients with type 2 diabetes

**DOI:** 10.1007/s00592-025-02626-9

**Published:** 2025-11-27

**Authors:** Riccardo Candido, Barbara Toffoli, Gabriele Baccichetto, Francesca Marchese, Silvia Carpenè, Sara Gaiotti, Bruno Fabris, Stella Bernardi

**Affiliations:** 1 Department of Medical Surgical and Health Sciences, University of Trieste, Cattinara Teaching Hospital, Strada di Fiume 447, 34149 Trieste, Italy; 2 SC Patologie Diabetiche, ASUGI (Azienda Sanitaria Universitaria Giuliano Isontina), Via Sai 7, 34128 Trieste, Italy; 3 SS Endocrinologia (Medicina Clinica), ASUGI (Azienda Sanitaria Universitaria Giuliano Isontina), Cattinara Teaching Hospital, Strada di Fiume 447, 34149 Trieste, Italy

**Keywords:** Oral semaglutide, GLP1R, Polymorphism, *rs6923761*, *rs761387*, Type 2 diabetes

## Abstract

**Aims:**

Glucagon-like peptide-1 receptor (GLP-1R) has become one of the most promising ligand-receptor systems to target for type 2 diabetes mellitus (T2DM) treatment. Over the last two decades, several GLP-1 receptor agonists (GLP-1RAs) have been developed and semaglutide is the first and only GLP-1RA available as an oral formulation*. GLP1R* single nucleotide polymorphisms may affect GLP-1R response to oral semaglutide. Here we aimed to evaluate the impact of *rs6923761* and *rs761387 GLP1R* polymorphisms on the response to oral semaglutide.

**Methods:**

This is a retrospective cohort study including adult patients with T2DM who had been treated with oral semaglutide for at least one year. Patients were enrolled between November 2023 and April 2024, and then genotyped.

**Results:**

We selected 210 adult patients with a median age of 71 years. Their median BMI was 29.1 kg/m^2^, HbA1c was 7.2% (55 mmol/mol), duration of diabetes was 12 years. After a median follow-up of 18 months, oral semaglutide reduced HbA1c by −0.3% (−3 mmol/mol), BMI by −1.1 kg/m2, SBP by −5 mmHg, total cholesterol by -8 mg/dL, triglycerides by -6.5 mg/dL. In addition, a reduction of ACR by −44.02 mg/g was observed in patients with baseline ACR > 30 mg/g, along with a decrease of liver transaminases in patients with baseline levels ≥ 35 U/L. Multivariate linear regression did not show any significant association between *rs6923761* or *rs761387 GLP1R* genotypes and changes in HbA1c, BMI, SBP and DBP.

**Conclusions:**

Our findings confirm the effectiveness of oral semaglutide in improving metabolic control and providing cardiorenal protection in different clinical scenarios. Conversely, they fail to show a clear benefit of *GLP1R* genotyping to guide treatment decisions, at least in patients with HbA1c < 7.5% (< 58 mmol/mol). Further studies are needed to confirm and extend our findings.

**Supplementary Information:**

The online version contains supplementary material available at 10.1007/s00592-025-02626-9.

## Introduction

The glucagon-like peptide-1 receptor (GLP-1R) is a key target for the treatment of type 2 diabetes mellitus (T2DM). GLP-1R signaling potentiates glucose-dependent insulin secretion, insulin biosynthesis, it inhibits glucagon secretion, slows gastrointestinal mobility and appetite, whereby it lowers glucose levels and body weight [1]. Despite reduced endogenous GLP1 levels in T2DM [2], GLP1-R function is preserved. As a result, GLP-1/GLP-1R axis has become one of the most promising ligand-receptor systems to target for therapeutic intervention in patients with T2DM [3], and over the last two decades, several GLP-1 receptor agonists (GLP-1RAs) have been developed. They include modified GLP-1 synthetic forms of the peptide exendin-4 (exenatide, exenatide extended-release, and lixisenatide), as well as modified GLP-1 analogs (liraglutide, dulaglutide, semaglutide) that allow for once-weekly administration. Semaglutide has 94% homology with native GLP-1, with three key modifications: an amino acid substitution at position 8 to protect against degradation, an acylation at position 26 with a C18 fatty di-acid chain, and an aminoacid substitution at position 34 to prevent fatty acid binding [4].

Semaglutide is the first and only GLP-1RA available as an oral formulation. This is made possible through its coformulation with SNAC (sodium N-[8-(2-hydroxybenzoyl)amino] caprylate), which protects the peptide from enzymatic degradation in the stomach and enhances its absorption [5]. The efficacy and safety of oral semaglutide were evaluated in the peptide-innovation-for-early-diabetes-treatment (PIONEER) randomised controlled trials (RCTs), which demonstrated improved glycemic control, weight loss, as well as favorable changes in cardiovascular risk factors [6]. Several real-world studies have confirmed the efficacy of oral semaglutide in terms of HbA1c and weight reduction as well as cardiovascular risk factor improvement [7, 8]. In particular, in prospective real-world studies HbA1c reduction was between -0.9% and -1.6% and in retrospective real-world studies HbA1c reduction was between -0.4% and -1.8% [7, 8]. In addition, the 14 mg daily dose of oral semaglutide consistently produced 3–4 kg of weight loss over 26 weeks across multiple PIONEER studies as well as in real-world studies [7, 8].

GLP-1R is encoded by the *GLP1R* gene, located on chromosome 6p21.2 [9]. The presence of naturally occurring nonsynonymous single nucleotide polymorphisms (SNPs) may lead to a change in *GLP1R* expression and to an impairment of GLP-1R signaling or ligand binding properties, ultimately affecting its response to GLP-1RAs [3, 10]. Overall, studies evaluating the impact of *GLP1R* SNPs on the response to GLP-1RAs are limited and somehow conflicting. One of the most well known *GLP1R* SNPs is the gene variant *rs6923761*, whose minor A allele frequency is 29% in White European individuals. This variant leads to the substitution of serine for glycine at position 168 (p.Gly168Ser, G > A). Initially, the *rs6923761* G > A variant was associated with larger weight reductions after liraglutide treatment [11, 12]. More recently, this variant has been linked to lower HbA1c reduction in T2DM patients [13]. Recent real-world data seem to confirm that carriers of the A allele have smaller reduction in HbA1c [14] but greater weight loss in response to GLP-1RAs [15]. Recently, also the *rs761387* A > G variant has been associated with glucose metabolism, as carriers of the minor G allele showed elevated postprandial glucose and GLP-1 levels [16], but its response to GLP-1RAs has not been investigated yet. Based on these premises, this retrospective real-world study aimed to evaluate the impact of *rs6923761* and *rs761387* polymorphisms on the response to oral semaglutide.

## Materials and methods

### Study design

This is a retrospective cohort study aiming to investigate the association between *GLP1R* gene variants *rs6923761*G > A and *rs761387*A > G and the response to oral semaglutide. Primary endpoint was HbA1c reduction. Secondary endpoints were body mass index (BMI), systolic blood pressure (SBP) and diastolic blood pressure (DBP) reduction.

This study was conducted in accordance with the Declaration of Helsinki, and the protocol was approved by the Institutional Review Board of the University of Trieste, Trieste, Italy (University of Trieste Comitato Etico di Ateneo #136 date 30/11/2023). Its design follows the recommendations of Strengthening the Reporting of Observational Studies in Epidemiology (STROBE) Statement [17], whose checklist is in Supplementary Table 1.

Eligible participants were adult patients (age > 18 years) with T2DM who were followed in the ASUGI Diabetes Center (S.C. Patologie Diabetiche, ASUGI, Trieste, Italy) and who had been treated with oral semaglutide for at least 12 months. Exclusion criteria were: (i) patients under the age of 18; (ii) absence of follow-up data; (iii) non-adherence to the prescribed medication; (iv) diagnosis of type 1 diabetes mellitus or gestational diabetes; and (v) lack of informed consent to participate in the study.

Based on these criteria, between November 2023 and April 2024 we consecutively identified 210 patients, who were fully informed on the purpose of the research and gave their written informed consent for the inclusion in this study. From electronic medical records we collected patient data regarding demographic characteristics, disease duration, medication (withdrawn and/or associated, if any), final dose of oral semaglutide, length of follow-up (from oral semaglutide introduction to last visit), as well as HbA1c, body weight, BMI, SBP and DBP, lipid panel, creatinine, albumin/creatinine ratio (ACR), aspartate aminotransferase (AST), alanine aminotransferase (ALT). Drug discontinuation and adverse events were also recorded.

### GLP1-R polymorphism

Among the subjects that were identified, a subgroup of 123 patients consented for genetic analyses. In these patients, a blood sample was collected for analysis of *GLP1R* SNPs. Genotyping of the *GLP1R* polymorphism was performed on peripheral blood mononuclear cells (PBMCs), as previously described [18]. PBMCs were isolated by density gradient centrifugation from anticoagulant-treated blood samples layered on Ficoll-Paque™ Plus solution (Cytiva, Marlborough, MA, USA). The mononuclear cells obtained were used to extract genomic DNA with the AllPrep DNA/RNA mini kit (Qiagen, Hilden, Germany), following the manufacturer’s instructions. The *GLP1R* polymorphisms (*rs6923761* and *rs761387*) were genotyped using a real-time TaqMan allelic discrimination assay (TaqMan SNP Assay; ThermoFisher Scientific Waltham, MA USA, Assay ID: C_25615272_20 (rs6923761); C_9616_10 (rs761387). *GLP1R* SNPs were selected based on the literature, as previously described. The distribution of allelic frequencies was analyzed by a χ^2^ test to show the potential deviation from the Hardy–Weinberg equilibrium.

### Statistical analysis

Sample-size was based on the literature [11, 14]. All statistical analyses were performed using the software “R” for statistical computing (version 4.4.0; R Development Core team, The R Foundation for Statistical Computing). Statistical significance was considered when p-value was < 0.05. Continuous variables were reported as median with interquartile range (IQR); categorical variables were expressed as absolute frequencies and percentages. The Shapiro–Wilk test was applied to continuous variables to check for distribution normality. Paired analysis (baseline – last follow up) were performed with the Wilcoxon test. Independent group comparisons were performed with Kruskal–Wallis test. The analysis of any association between *GLP1R* polymorphisms and response to therapy was performed with linear regression models in order to adjust for confounders variables. In these models, either HbA1c reduction, or BMI reduction, or SBP reduction, or DBP reduction was the dependent variable, adjusted for baseline HbA1c, baseline BMI, age, sex, dose of oral semaglutide, use of SGLT2inhibitor. Baseline SBP or baseline DBP were also included when the dependent variable was either SBP reduction or DBP reduction, respectively.

## Results

### Population characteristics

A total of 210 patients with T2DM treated with oral semaglutide were enrolled, and their baseline characteristics are reported in Table 1. The population had a median age of 71 years (IQR 64; 78) and 58% of them were males (121/210). Their median BMI was 29.1 kg/m^2^ (IQR 26.4; 33.2). Median HbA1c level was 7.2% (IQR 6.6; 8.0) or 55 mmol/mol (QR 49; 64). Duration of diabetes was 12 years (IQR 6; 21). Oral semaglutide was started at the dose of 3 mg for 4 weeks, and then, based on glucose control and gastrointestinal tolerability, it was increased up to the final dose of 7 mg in 80/210 patients (38.1%) and 14 mg in 116/210 patients (52.2%). The median follow-up period was 18 months (IQR 12; 18). Of note, 191/210 patients (91%) were prescribed oral semaglutide in addition to another glucose-lowering drug, in particular 133/210 (63%) were taking metformin; 108/210 (51%) were taking SGLT2 inhibitors and 69/210 (33%) were taking insulin. Overall, treatment was suspended in 49/210 (23%) patients, mostly because of mild gastrointestinal effects. Only 4/210 (1.9%) cases of hypoglicemia were recorded.

**Table 1:** Characteristics of the entire cohort and GLP-1R genotype subgroups at baseline

Variable	All patients (*n* = 210)	*GLP-1R* (*rs6923761*) GG Genotype (*n* = 49)	*GLP-1R* (*rs6923761*) GA Genotype (*n* = 57)	*GLP-1R* (*rs6923761*) AA Genotype (*n* = 17)	*GLP-1R* (*rs761387*) AA Genotype (*n* = 98)	*GLP-1R* (*rs761387*) AG + GG Genotype (*n* = 25)
Age (years)	71 (64–78)	68 (62–75)	69 (64–76)	71 (66–76)	68 (62–76)	73 (69–76)
Sex M/F (n; %)	121/89 (58%/42%)	30/19 (61/39%)	40/17 (70/30%)	8/9 (47/53%)	65/33 (66–34%)	13/12 (52–48.%)
Diabetes duration (years)	12 (6–21)	14 (10–23)	11 (5–16)	4 (3–9)*	12 (8–19)	9 (4–19)
HbA1c (%)	7.2 (6.6–8.0)	7.6 (6.9–8.6)	7.1 (6.6–7.6)	7.0 (6.6–8.7)	7.2 (6.6–8.2)	7.3 (6.5–8.0)
HbA1c (mmol/mol)	55 (49–64)	60 (52–70)	54 (49–60)	53 (49–72)	55 (49–66)	56 (48–64)
BMI (kg/m^2^)	29.1 (26.4–33.2)	28.4 (25.9–32.7)	31.4 (27.2–34.4)	30.0 (28.4–32.1)	30.0 (26.8–34.2)	31.1 (26.8–33.9)
Dose of oral semaglutide (mg)	14 (7–14)	14 (7–14)	14 (7–14)	14 (7–14)	14 (7–14)	14 (7–14)
Follow-up duration (months)	18 (12–18)	18 (12–18)	18 (12–18)	18 (12–18)	18 (12–18)	18 (12–18)
SBP (mmHg)	130 (120–140)	130 (120–140)	135 (120–140)	140 (127–149)	135 (120–142)	130 (123–136)
DBP (mmHg)	80 (70–80)	70 (70–80)	80 (70–87)*	80 (72–85)	80 (70–85)	72 (70–80)
Total cholesterol (mg/dL)	153 (125–177)	153 (122–168)	148 (128–170)	155 (122–215)	150 (125–180)	153 (122–159)
HDL (mg/dL)	49 (41–59)	47 (37–57)	48 (40–58)	52 (43–60)	48 (39–57)	46 (42–63)
LDL (mg/dL)	68 (51–101)	72 (55–99)	67 (50–82)	87 (49–127)	68 (51–104)	68 (50–84)
Triglycerides (mg/dL)	127 (99–176)	109 (90–164)	143 (102–192)	129 (103–191)	141 (96–185)	121 (94–196)
ACR (mg/g)	10.0 (4.3–25.9)	6.45 (2.97–22.12)	9.2 (5.2–21.2)	6.5 (3.5–13.0)	8.2 (4.4–22.0)	6.2 (3.5–10.7)
GFR (mL/min)	77 (61–94)	80 (65–99)	80 (64–98)	80 (66–90)	81 (66–100)	77 (57–89)
AST (U/L)	21 (17–27)	21 (17–26)	20 (17–27)	21 (16–28)	20 (16–26)	24 (18–28)
ALT (U/L)	21 (16–30)	20 (17–31)	23 (17–29)	22 (17–31)	21 (17–29)	24 (17–35)

Data are presented as median (IQR). *p < 0.05 was considered statistically significant vs GG genotype (rs6923761)

### Response to oral semaglutide

**Fig. 1:**
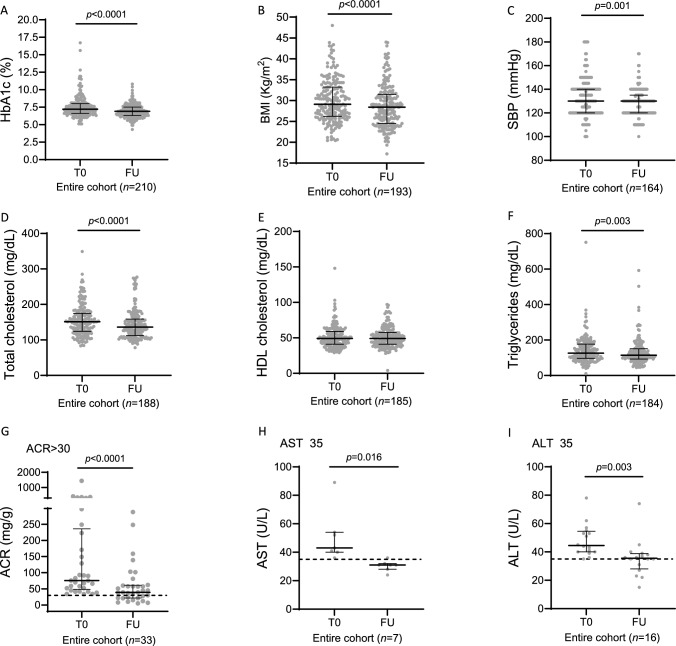
Effects of oral semaglutide on metabolic and cardiovascular parameters. Paired comparison of baseline vs last follow-up was performed with Wilcoxon-matched signed rank pairs for the following variables: **A** HbA1c; **B** BMI; **C** SBP; **D** total cholesterol; **E** HDL cholesterol; **F** tryglicerides; **G** ACR; **H** AST; **I** ALT. Data are reported as median values ± IQR

First, we looked at the overall response to oral semaglutide after a median follow-up of 18 months. As shown in Fig. 1, oral semaglutide significantly reduced HbA1c by -0.3% (IQR -1; 0.2) or -3 mmol/mol (IQR -11; 2), BMI by -1.1 kg/m^2^ (IQR -2.2; -0.3), SBP by -5 mmHg (IQR -15; 5), total cholesterol by -8 mg/dL (IQR -29; 9) and triglycerides by -6.5 mg/dL (IQR -32; 10.25) in the entire cohort. ACR was significantly reduced by -44.2 mg/g in the subgroup of patients with ACR > 30 mg/g. Likewise, AST and ALT were also significantly reduced in the subgroup of patients with transaminase baseline levels ≥ 35 U/L.

### Polymorphism distribution

Genetic analysis was performed on 123 patients for both *rs6923761* and *rs761387 GLP1R* polymorphisms. The Hardy–Weinberg equilibrium test comparing the observed allele with the expected ones, showed that the minor allele frequencies (MAF) that we found were in line with those of the general population. MAF of *rs6923761* was 0.37 and MAF of *rs761387* was 0.11. Allele frequency and genotype distributions are shown in Table 2.

**Table 2:** Distribution of observed allele frequencies

SNP	Nucleotide changes	Genotype	N (%)	MAF%	pHWE
*GLP1R* *rs6923761*	NM_002062.5:c.502G > A	GG	49 (39.9%)	37%	0.23
		GA	57 (46.3%)		
		AA	17 (13.8%)		
*GLP1R* *rs761387*	NM_002062.5:c.884 + 43A > G	AA	98 (79.7%)	11%	0.74
		AG	23 (18.7%)		
		GG	2 (1.6%)		

MAF, minor allele frequency; pHWE, p-value of the χ^2^ test for Hardy–Weinberg equilibrium

The impact of *GLP1R rs6923761* variant was analyzed taking into account both the co-dominant and dominant inheritance models, because 49/123 patients (39.9%) carried the wild-type GG variant, 57/123 patients (46.3%) carried the heterozygous mutated GA variant, and 17/123 patients (13.8%) carried the homozygous mutated AA variant. The *rs761387* variant was analyzed taking into account the dominant inheritance model only, as 98/123 patients (79.7%) carried the wild-type AA variant, 23/123 patients (18.7%) carried the heterozygous mutated AG variant, and only 2/123 patients (1.6%) carried the homozygous mutated GG variant.

### Impact of *GLP1R* gene variants on the response to oral semaglutide

As shown in Table 1, there were no differences in terms of baseline characteristics between the subgroups based on *GLP1R* genotype, except for a significantly shorter duration of diabetes in the *GLP1R rs6923761* AA genotype (p < 0.05), and a higher blood pressure in the *GLP1R rs6923761* GA and AA genotype (p < 0.05).

Table 3 reports changes in HbA1c, BMI, SBP and DBP. Only DBP appeared significantly reduced after 18 months of oral semaglutide in subjects with the GLP1R *rs6923761 GA* + *AA* genotypes. Otherwise, there were no other differences between genotypes.

**Table 3:** Association of GLP1R SNPs on changes in metabolic and cardiovascular parameters

SNP	Genotype	N	Change of HbA1c (%)	p-value
*GLP-1R rs6923761*	GG	49	−0.50 (−1.35; 0.175)	
	GA	57	−0.30 (−0.80; 0.175)	
	AA	17	−0.60 (−1.20; −0.30)	0.291
	GA + AA	74	−0.40 (−0.95; 0.05)	0.821
*GLP-1R rs761387*	AA	98	−0.40 (−0.975; 0.175)	
	AG + GG	25	−0.50 (−1.40; 0.0)	0.529

			Change of HbA1c (mmol/mol)	
*GLP-1R rs6923761*	GG	49	−6 (−15.5; 2)	
	GA	57	−3 (−9; 2)	
	AA	17	−7 (−13; −3)	0.291
	GA + AA	74	−4 (−11; 0)	0.821
*GLP-1R rs761387*	AA	98	−4 (−11; 2)	
	AG + GG	25	−5 (−15; 0)	0.529

			Change of BMI (Kg/m^2^)	
*GLP-1R rs6923761*	GG	49	−1.4 (−2.1; −0.72)	
	GA	57	−0.80 (−2.25; −0.38)	
	AA	17	−1.70 (−2.75; −1.0)	0.219
	GA + AA	74	−1.0 (−2.55; −0.40)	0.266
*GLP-1R rs761387*	AA	98	−1.35 (−2.32; −0.57)	
	AG + GG	25	−0.50 (−2.20; 0.30)	0.155

			Change of SBP (mmHg)	
*GLP-1R rs6923761*	GG	49	0.0 (−10.0; −5.0)	
	GA	57	−5.0 (−20.0; −10.0)	
	AA	17	−10.0 (−20.0; −5.0)	0.063
	GA + AA	74	−10.0 (−20.0; 0.0)	0.108
*GLP-1R rs761387*	AA	98	−7.0 (−18.5; −5.0)	
	AG + GG	25	0.0 (−5.0; 0.0)	0.460

			Change of DBP (mmHg)	
*GLP-1R rs6923761*	GG	49	0.0 (−5.0; 10.0)	
	GA	57	−5.0 (−15.0; 0.0)	
	AA	17	−10.0 (−13.75; 0.0)	0.013
	GA + AA	74	−5.0 (−15.0; 0.0)	0.005
*GLP-1R rs761387*	AA	98	−5.0 (−10.0; −2.0)	
	AG + GG	25	0.0 (−5.0; 5.0)	0.245

Multivariate linear regressions were performed to evaluate if *GLP-1R* gene variants had an impact on oral semaglutide response in terms of HbA1c, BMI, and blood pressure reduction, taking into account possible confounders. Multivariate linear regressions did not show any significant association between *rs6923761* or *rs761387 GLP1R* genotypes and changes in HbA1c, BMI, SBP and DBP. By contrast, HbA1c reduction was significantly associated with baseline HbA1c (β-estimate −0.75; 95%CI −0.89; −0.61; P < 0.0001). BMI reduction was significantly associated with baseline BMI (β-estimate −0.18; 95%CI −0.27; −0.04; P < 0.009) and dose of oral semaglutide (β-estimate 4.19; 95%CI −0.06; 8.44; P < 0.053). SBP reduction was significantly associated with baseline SBP (β-estimate −0.69; 95%CI −0.84; −0.54; P < 0.0001). DBP reduction was significantly associated with baseline DBP (β-estimate −0.72; 95%CI −0.92; −0.52; P < 0.0001) and baseline HbA1c (β-estimate 1.28; 95%CI 0.23; 2.34; P < 0.017).

## Discussion

Oral semaglutide is the first oral GLP-1RA approved for managing T2DM. In this retrospective study, we aimed to evaluate the impact of *rs6923761* and *rs761387* on the response to this drug. For this purpose, we selected 210 patients with T2DM taking oral semaglutide and we genotyped 123 of them. Our patients were more than 70 year-old and had baseline HbA1c of 7.2% (55 mmol/mol). To our knowledge, this is one of the oldest populations [19] and with the lowest baseline HbA1c levels, among the recruited T2DM patients taking oral semaglutide. In addition, their BMI was 29.1 kg/m^2^ and 91% of our patients were prescribed another glucose-lowering drug, most often metformin (63%) or SGLT2 inhibitors (51%).

First of all, our data show that after a follow-up of 18 months, oral semaglutide reduced HbA1c by −0.3% (−3 mmol/mol), BMI by −1.1 kg/m^2^, and SBP by −5 mmHg. In addition, it reduced total cholesterol by −8 mg/dL, tryglicerides by -6.5 mg/dL, and ACR by −44.02 mg/g in the subgroup with ACR > 30 mg/g. Also liver transaminases were significantly reduced in the subgroup of patients with baseline levels ≥ 35 U/L. As compared with our previous results demonstrating the efficacy of oral semaglutide in terms of HbA1c and BMI reduction after 6 months [20], here we show that its effects extend to 18 months of follow-up. When looking at HbA1c and BMI reduction, it has to be kept in mind that the degree of their reduction depends on their baseline values [20]. For example, in the IGNITE study, the patients with baseline HbA1c > 9% had mean HbA1c reduction of −2.1% [21]. As for blood pressure and lipids, our data align with a recent systematic review showing that across five high-quality studies (~ 5,100 participants), oral semaglutide led to reductions in both systolic blood pressure (≈ − 2.6 to − 12.7 mmHg) and lipids (total cholesterol − 8.8 to − 22.2 mg/dL; LDL-C − 7.6 to − 18.0 mg/dL; triglycerides − 11 to − 40 mg/dL) [22]. In addition, they align with the SOUL trial, showing that oral semaglutide was associated with a 14% reduction in the risk of major adverse cardiovascular events than placebo (HR 0.86; 95% CI 0.77–0.96)[23]. Consistent with these changes, in this study, we found a significant reduction of ACR in the subgroup of patients with baseline values > 30 mg/g, which is in line with the report by Yanai [24] and Moreno-Perez [25]. Last, we also found that oral semaglutide significantly reduced liver transaminases, in patients with baseline levels ≥ 35 U/L. This is consistent with the report by Arai et al. showing that oral semaglutide significantly reduced liver transaminases as well as liver fibrosis marker in subjects with metabolic-associated steatotic liver disease and T2DM after 24 months of therapy [26]. Subsequent studies have confirmed that oral semaglutide is effective in ameliorationg liver disease [27].

With respect to the impact of *GLP1R* polymorphisms on the response to oral semaglutide, we focused on the *rs6923761* and *rs761387 GLP1R* variants. Several studies indicate that the *rs6923761* G > A variant could be a potential genetic biomarker of treatment response to GLP-1RAs in Caucasian populations [12, 13, 28]. For instance, a study on healthy participants showed that carriers of the minor A allele (Gly168Ser) had lower insulin secretion after an infusion of GLP-1[28]. Also in T2DM patients, carriers of the minor A allele exhibited a reduced glycemic response to gliptin [29]. Most importantly, a recent genome-wide analysis on 4571 patients showed that carriers of the minor A allele had lower HbA1c reduction after treatment with GLP-1RAs (liraglutide, exenatide, albiglutide, dulaglutide) [13]. Likewise, Tonin et al. found that the minor A allele was associated with lower glycemic response to subcutaneous GLP-1RAs (semaglutide, liraglutide, dulaglutide). On the other hand, it has been suggested that also the *rs761387 A* > *G* variant could impact on the treatment response to GLP1-RAs. In particular, a recent work has shown that the minor G allele of the *rs761387* variant was associated with altered insulin signaling in 868 subjects with T2DM or at risk for developing diabetes, as during the OGTT the G allele was associated with higher glucose levels [16].

In our study, we found no association between the *rs6923761* or the *rs761387 GLP1R* variant and changes in HbA1c, BMI, SBP or DBP. Our findings are in line with those reported by Eghbali and de Luis [11], who showed no associations between the *rs6923761* minor A allele and glycemic response to liraglutide [30], in populations that were younger (age between 53 and 61 years) than ours, with higher HbA1c (> 8%) and higher BMI > 30 kg/m^2^. However, our findings differ from other works [13, 14, 29]. For instance, Tonin et al. showed that in 70 patients with T2DM (whose median age was 66 years, HbA1c 6.8%, BMI 33 kg/m^2^) receiving subcutaneous GLP-1RAs, the *rs6923761* minor A allele was associated with a smaller reduction in HbA1c (i.e. -1.4% in GG as compared to -0.8% in GA + AA genotype) [14]. Therefore, in our study, the lack of association between *GLP1R* genotype and HbA1c or BMI reduction could be due to several reasons. First, our population was older (70 years) and had lower BMI (29.1 kg/m^2^) as compared to all the previously cited works. Second, in the previous studies, the GLP-1RAs that were prescribed included liraglutide, dulaglutide and semaglutide, while our patients were treated with oral semaglutide. Oral semaglutide has a different pharmacokinetic profile as compared to all subcutaneous GLP-1RAs [30], which can be further modified if the drug is not taken correctly [4], moreover, it is unknown whether SNAC absorption might modulate its effects on *GLP1R* variants. Third, although the sample size did not differ from the number of patients recruited by Tonin [14], as well as other Authors [11, 15], it should have been larger for a median HbA1c change of 0.3% and a median BMI change of −1.1 kg/m^2^. Having said that, it has been argued that T2DM is a heterogeneous polygenic disease, with many patient characteristics interacting and diluting the effect of any particular SNP, such that the integration of pharamacogenetic principles into precision diabetology is highly complex [31]. Therefore, predictions of drug efficacy based on genotype have a degree of uncertainty and need to take into account various metabolic and behavioral factors.

In conclusion, this study confirms oral semaglutide efficacy on several cardio-metabolic parameters, including not only HbA1c, but also BMI, SBP, lipids, ACR and transaminases. In this study, we did not find an association between GLP1R *rs6923761* and *rs761387* polymorphisms and the response to oral semaglutide in terms of HbA1c reduction as well as BMI, SBP and DBP reduction. Our findings confirm the effectiveness of oral semaglutide in improving metabolic control and providing cardiorenal protection in different clinical scenarios. Conversely, they fail to show the clear benefit of *GLP1R* genotyping to guide treatment decisions, at least in patients with HbA1c < 7.5% (< 58 mmol/mol). Further studies are needed to confirm and extend our findings.

## Supplementary Information

Below is the link to the electronic supplementary material.

Supplementary file1 (DOC 102 KB)

## Data Availability

The raw data supporting the conclusions of this article will be made available by the authors upon reasonable request.
